# 2018 Korean Society of Hypertension Guidelines for the management of hypertension: part II-diagnosis and treatment of hypertension

**DOI:** 10.1186/s40885-019-0124-x

**Published:** 2019-08-01

**Authors:** Hae-Young Lee, Jinho Shin, Gheun-Ho Kim, Sungha Park, Sang-Hyun Ihm, Hyun Chang Kim, Kwang-il Kim, Ju Han Kim, Jang Hoon Lee, Jong-Moo Park, Wook Bum Pyun, Shung Chull Chae

**Affiliations:** 1 Department of Internal Medicine, Seoul National University College of Medicine, Seoul, Korea; 2 Department of Internal Medicine, Hanyang University College of Medicine, Seoul, Korea; 3 Department of Internal Medicine, Yonsei University, Seoul, Korea; 4 Division of Cardiology, Department of Internal Medicine, College of Medicine, The Catholic University of Korea, Seoul, Korea; 5 Department of Internal Medicine, Seoul National University, Seoul National University Bundang Hospital, Seongnam, Korea; 6 Department of Internal Medicine, School of Medicine, Chonnam University, GwangJu, Korea; 7 Division of Cardiology, Department of Internal Medicine, Kyungpook National University School of Medicine, Daegu, Korea; 8 Department of Neurology, Nowon Eulji Medical Center, Eulji University, Seoul, Korea; 9 Cardiovascular Center, Seoul Hospital, Department of Internal Medicine, Ewha Womans University School of Medicine, Seoul, Korea

**Keywords:** Blood pressure, Measurement, Cardiovascular risk, Guidelines, Hypertension, Lifestyle, Antihypertensive treatment

## Abstract

The standardized techniques of blood pressure (BP) measurement in the clinic are emphasized and it is recommended to replace the mercury sphygmomanometer by a non-mercury sphygmomanometer. Out-of-office BP measurement using home BP monitoring (HBPM) or ambulatory BP monitoring (ABPM) and even automated office BP (AOBP) are recommended to correctly measure the patient’s genuine BP. Hypertension (HTN) treatment should be individualized based on cardiovascular (CV) risk and the level of BP. Based on the recent clinical study data proving benefits of intensive BP lowering in the high risk patients, the revised guideline recommends the more intensive BP lowering in high risk patients including the elderly population. Lifestyle modifications, mostly low salt diet and weight reduction, are strongly recommended in the population with elevated BP and prehypertension and all hypertensive patients. In patients with BP higher than 160/100 mmHg or more than 20/10 mmHg above the target BP, two drugs can be prescribed in combination to maximize the antihypertensive effect and to achieve rapid BP control. Especially, single pill combination drugs have multiple benefits, including maximizing reduction of BP, minimizing adverse effects, increasing adherence, and preventing cardiovascular disease (CVD) and target organ damage.

These Korean Society of Hypertension guidelines for the management of hypertension defined levels of evidence as below: A, Data derived from multiple randomized controlled trials or meta-analysis; B, Data derived from a single randomized controlled trials or non-randomized clinical trials; C, Experts’ opinion or data derived from limited evidences. Also classes f recommendations were defined as below: Class I, Evidence and/or general agreement that a given treatment or procedure is beneficial, useful, and effective. Therefore it should be performed; Class IIa, Conflicting evidence and/or a divergence of opinion about the usefulness/efficacy of the given treatment or procedure exists. However, in general, weight of evidence/opinion is in favor of usefulness/efficacy. Therefore it is reasonable to be performed; Class IIb, Usefulness/efficacy is less well established by evidence/opinion. Therefore it may be considered; Class III, Evidence or general agreement that the given treatment or procedure is not beneficial and in some cases may be harmful. Therefore it is not recommended.

## Chapter I. clinical evaluation

### Blood pressure measurement

Accurate measurement of BP is essential for the diagnosis, treatment, and prognostication of individuals with high BP. BP varies according to the environment, body part, and clinical setting of measurement. Therefore, the measurement should be repeated and a standard method should be used (Table 1). All adults who are over 40 years old, obese, or have family history of HTN should measure blood pressure annually. In patients with prehypertension (130–139/80–89 mmHg), BP should also be measured annually.

**Table 1:** Blood pressure measurement

• Rest for 5 or more minutes in a quiet, appropriate environment	
• Avoid smoking, alcohol or caffeine before measurement	
• Measure 2 or more times at 1- to 2-min intervals at a single visit	
• Use a cuff with a bladder at least 40% of the arm circumference wide; 80% of arm circumference long (a standard bladder for adults: 13 cm wide; 22–24 cm long)	
• Maintain the upper arm cuff at the heart level	
• Inflate the cuff rapidly and deflate slowly at a speed of 2 mmHg per heart beat	
• Identify the blood pressure as the systolic blood pressure at the first Korotkoff sound; the blood pressure as the diastolic blood pressure at the fifth Korotkoff sound	
• Consider the blood pressure as the diastolic blood pressure at the fourth Korotkoff sound in pregnancy, arteriovenous shunt, and chronic aortic insufficiency	
• Measure blood pressure in both arms on the initial visit; subsequently use the arm of higher pressure to measuring blood pressure	
• Measure blood pressure in legs to exclude peripheral arterial disease, when pulses in the lower extremities are weak	
• Repeating the measurement three or more times to estimate the average systolic and diastolic pressure in case of arrhythmia	
• Measure blood pressure after 1- and 3-min standing in elderly persons and persons with diabetes and suspected orthostatic hypotension	

#### Measurement of the office or clinic blood pressure

**Table:** 

**Recommendations**	**Class**	**Level**	**References**
• A mercury sphygmomanometer is recommended to be replaced by a non-mercury sphygmomanometer.	**IIa**	**C**	[1]

In the office or clinic, the auscultatory method of measuring BP using a stethoscope is the current standard measurement method. BP measuring devices used for the office BP measurement include a mercury sphygmomanometer, an aneroid sphygmomanometer, and an electronic sphygmomanometer. Electronic sphygmomanometers comprise auscultation and vibration method equipment. The mercury sphygmomanometer will be banned by 2020 because of the risk of environmental mercury pollution. Recently, the non-mercury sphygmomanometer equipped with various electronic pressure gauges has been certified and is commercially available so that BP can be measured by auscultation or by the oscillometric method instead of by mercury BP monitoring (http://www.dableducational.org).

For BP measurement based on the auscultation method, the differences between mercury and non-mercury sphygmomanometers are negligible and furthermore, non-mercury sphygmomanometers are less subject to observed eye-level error. However, oscillometric method might be less accurate in the elderly, pregnant women, children, or subjects with arrhythmia. After the patient rests for five or more minutes, the BP is measured by the auscultation method. The measurement is reported two or more times (Table 1).

A cuff with an appropriately sized bladder should be used. The standard bladder for adults is 13 cm wide and 22–24 cm long. The use of a bladder with a width of at least 40% of the circumference of the arm and a length of 80–100% of the circumference of the arm is recommended. A cuff of appropriate size as recommended by the manufacturer should be used.

In the case of the automatic sphygmomanometers of vibration method, a cuff of the size recommended in the instruction manual should be used. A cuff that is too small can produce an artificially high BP reading, whereas a cuff that is too large can produce an artificially low BP reading. If the pulses of the lower extremities are weak, BP is measured in the legs to exclude the possibility of peripheral arterial disease (PAD). The upper arm cuff is applied to the ankle and auscultation is performed on the dorsalis pedis or posterior tibial artery. A large cuff can be applied to the thigh, using a bladder that is 20% wider than the diameter of the thigh (15–18 cm). Auscultation is performed on the posterior tibial artery. Because the measured value varies widely in case of arrhythmia, the BP values should be averaged with more than three times of measurement [2].

#### Home blood pressure measurement

**Table:** 

**Recommendations**	**Class**	**Level**	**References**
• Home BP measurement is recommended to diagnose sustained HTN, white coat HTN and masked HTN and to estimate prognosis.	**I**	**A**	[3]
• For accurate home BP measurements, accurate measurement method should be educated to all patients.	**I**	**C**	[4]

BP outside the office (out-of-office) can be measured by home BP measurement and ABPM. Recently, electronic sphygmomanometers using the vibration method have been widely used for their convenience and accuracy. Out-of-office BP measurement provides better prognostic information than does office BP measurement alone [3]. Home BP measurement is becoming increasingly important in not only for diagnosis but also for management of HTN. However, since home BP should be measured in a standardized way, patients should be educated on accurate measurement methods as shown in Table 2 (http://www.koreanhypertension.org/sense/family).

**Table 2:** Measurement of home blood pressure monitoring

• Use an upper arm cuff	
• Time of measurement should be	
1. Morning: within 1 h after waking up, after urination, before taking antihypertensive drugs, before breakfast, after a 1–2 min rest in a seated position 2. Night: before retiring, after a 1–2 min rest in a seated position 3. Other conditions if necessary	
• Frequency of measurement: one to three times per occasion	
• Period of measurement: as long as possible; 1 week or more for the diagnosis of hypertension; over at least 5–7 days immediately preceding the visit during follow-up of treatment	

Since the finger sphygmomanometer may be inaccurate, the upper arm sphygmomanometer should be used. The wrist sphygmomanometer should be allow the positioning of the cuff at the height of the heart, which might be useful in measuring BP in very obese patients, especially in the upper arm. White coat HTN refers to the untreated condition in which BP is elevated in the office, but is normal when measured by ABPM, home BP measurement, or both. Conversely, masked HTN refers to untreated patients in whom BP is normal in the office, but is elevated when measured by ABPM or home BP measurement (Table 3).

**Table 3:** Criteria for definition of hypertension with different methods of measurement

Category	Systolic blood pressure (mmHg)	Diastolic blood pressure (mmHg)
Clinic or office blood pressure	≥140	≥90
Ambulatory blood pressure		
24-h	≥130	≥80
Day	≥135	≥85
Night	≥120	≥70
Home blood pressure	≥135	≥85
Automated office blood pressure	≥135	≥85

Home BP measurement is recommended to diagnose sustained HTN, white coat HTN and masked HTN and to estimate prognosis. There is also evidence that home HTN monitoring may have a beneficial effect on medication adherence and BP control [5, 6].

Generally, home BP is lower than office BP. HTN is regarded as home BP is defined as 135/85 mmHg or more. “Morning HTN” is defined as BP measured in the morning is higher than 135/85 mmHg and higher than BP measured before sleep. When diagnosing HTN by HBPM, it is recommended to measure BP for more than 5 days a week, 1 to 3 times in the morning and night. After removing the BP value of the first day of the evaluation, the values are averaged. In the morning, it is recommended to measure BP within 1 h of waking up, after voiding the bladder, and before taking antihypertensive medication. In the evening, it is recommended to measure BP before going to bed. When HTN is first diagnosed, a seven-day average BP value is recommended. Recommendations of home BP measurement are as shown in Table 2 [7].

#### Ambulatory blood pressure measurement

**Table:** 

**Recommendations**	**Class**	**Level**	**References**
•Ambulatory blood pressure monitoring can be used for a number of clinical indications, such as identifying white coat and masked HTN, quantifying the effects of treatment, and estimating prognosis.	**IIa**	**A**	[8, 9]

Ambulatory BP measurement provides information on the BP during the daytime, nighttime, and specific periods, for example, early morning. Measurement of BP is performed at 15–30 min intervals over a 24-h period. Ambulatory BP measurement provides better prognostic information than does BP measurement in the clinic. The mean daytime ambulatory BP criterion for HTN is the same as for home BP (i.e., 135/85 mmHg or more) (Table 3). Ambulatory BP measurement is useful in clinical conditions such as white coat HTN, masked HTN, resistant HTN, labile HTN, HTN in pregnancy, and autonomic dysfunction and also when accurate measurement of BP is required for risk assessment or for the precise evaluation of BP lowering efficacy [10]. A morning surge is considered to be a risk factor for CVD, particularly stroke [11]. BP shows a diurnal rhythm, being higher during waking hours and lower during sleep. Normally, the average BP is 10–20% lower at nighttime than during the day (dipper). A difference of less than 10% (non-dipper) or an increase in the nighttime BP relative to the daytime BP (riser) is associated with increased risk of death, myocardial infarction, and stroke [12]. A decrease of 20% or more (extreme dipper) may be associated with increased risk for ischemic stroke and atherosclerosis [13]. Risers may have autonomic dysfunction and are at increased risk for hemorrhagic stroke [14]. When measuring ambulatory BP, it is important to instruct the patient to engage in his or her ordinary daily activities but to avoid strenuous exercise and also to hold the arm still and in extension during cuff inflation. It is also necessary to educate the patient on how to keep a diary and how to turn off the monitoring device.

#### Automated office blood pressure measurement

**Table:** 

**Recommendations**	**Class**	**Level**	**References**
•Automated office blood pressure can be measured to exclude white coat HTN.	**IIa**	**B**	[15]

Autimate office blood pressure (AOBP) is measured by setting the measurement time of the automatic sphygmomanometer in advance, after a 5-min rest in room alone without medical staff, and measuring the average value of BP three times at 1-min intervals. The reference value for HTN diagnosis is similar to home BP and daytime ambulatory BP, however the relationship between BP readings obtained with conventional office BP measurement and unattended office BP measurement remains unclear [16–18].

The AOBP measurement may reduce the white coat effect and should be considered when it is difficult to measure home BP or ambulatory BP, as it is useful for repeated measurement; nevertheless, it is disadvantageous for diagnosing masked HTN.

#### Central blood pressure measurement

Approximately 80% of the brachial arterial pressure can be attributed to the central arterial pressure. In addition, the central BP has been reported to be more closely correlated with target organ damage or prognosis. Although it might be useful for patients with significant differences between the central arterial pressure and brachial artery pressure, it is not usually recommended to replace conventional brachial BP [19, 20].

### Evaluation of the patient

Diagnosis and examination are aimed at: 1) differentiating primary and secondary HTN; 2) evaluating the severity of HTN; 3) identifying CV risk factors and lifestyle issues; and 4) searching for CVD, comorbidity, or subclinical organ damage that could affect the choice of treatment.

#### Symptoms and signs

Patients with HTN most often have no specific symptoms of BP elevation. HTN is usually found incidentally or else it is detected in conjunction with symptoms of hypertensive CVD or an underlying secondary cause of HTN. Headache is often considered a symptom of HTN, however, there is no significant correlation between BP and headache except in cases of severe HTN. Headache accompanied by HTN is commonly localized at the back of the head, occurs early in the morning upon awakening, and subsides spontaneously during the day. Some patients with HTN exhibit general, non-specific symptoms such as dizziness, palpitation, fatigue, and sexual dysfunction. The symptoms of hypertensive CVD are hematuria, blurred vision, dizziness due to transient ischemic attack, angina, and shortness of breath due to heart failure. In some rare cases, chest pain due to aortic dissection or aortic aneurysm can occur. Patients with secondary HTN can have specific symptoms and signs suggestive of the underlying causes. For example, patients with sleep apnea syndrome may experience early morning headache, excessive daytime sleepiness, depression, reduced concentration, and nocturnal shortness of breath. Patients with primary aldosteronism may have polyuria, polydipsia, and episodes of muscle weakness. Patients with Cushing’s syndrome may have weight gain and emotional instability and those with pheochromocytoma, episodic headache, palpitation, sweating, and orthostatic hypotension.

#### Medical history

The medical history includes: 1) personal history of present illness, past history, and family history; 2) symptoms and signs suggestive of secondary causes of HTN; 3) symptoms and signs of target organ damage; 4) CV risk factors; 5) concomitant diseases; 6) lifestyle factors such as diet, smoking, alcohol consumption, physical inactivity, exercise, sleep, and personality/psychological status; 7) duration and previous level of HTN, previous treatment and its results, and adverse effects of antihypertensive therapy; 8) use of nonsteroidal anti-inflammatory drugs, oral contraceptives, herbs, and other drugs; and 9) socioeconomic status.

#### Physical examination

Physical examination of hypertensive patients includes 1) measurement of BP in both arms as well as the pulse rate at the first visit; 2) measurement of height and weight to calculate the body mass index (BMI) as well as measurement of waist circumference; 3) auscultation for bruits over the carotid arteries, abdomen, and femoral arteries; 4) palpation of the thyroid; 5) examination of the heart and lungs; 6) examination of the abdomen for kidney enlargement, masses, bladder distension, and abnormal aortic pulsation; 7) examination of the lower extremities for edema and palpation of the pulses; and 8) neurological examination. Waist circumference is measured with a tape measure at the level midway between the lowest rib and the iliac crest with the patient in a standing position, at the end of normal expiration, with the abdomen exposed and without compression of the abdominal skin.

#### Laboratory examinations

**Table:** 

**Recommendations**	**Class**	**Level**	**References**
• It is recommended that the routine laboratory tests should be evaluated at the first visit and annually.	**IIa**	**C**	

Laboratory examinations are performed to identify additional CV risk factors, secondary causes of HTN, subclinical organ damage, and concomitant diseases. Routine laboratory tests should be performed before antihypertensive treatment. Other recommended and extended tests may be performed if necessary (Table 4).

**Table 4:** Laboratory examination

Routine tests	
12-leads electrocardiogram (ECG)	
Urinalysis – proteinuria, hematuria, glucosuria	
Hemoglobin, hematocrit	
K+, creatinine, estimated glomerular filtration rate (eGFR)^a^, uric acid,	
Fasting glucose, lipids [total cholesterol, high-density lipoprotein (HDL)-cholesterol, low-density lipoprotein (LDL)-cholesterol, triglyceride]	
Chest X-ray	
Microalbuminuria: albumin/creatinine (in random urine sample)	
Recommended tests	
75 g oral glucose tolerance test or hemoglobin A1c (if fasting glucose ≥100 mg/dL)	
Echocardiogram	
Carotid ultrasound: plaque	
Ankle-brachial blood pressure index	
Pulse wave velocity	
Fundoscopy (mandatory in diabetes)	
24-h urine protein excretion	
Extended tests	
Search for sub-clinical organ damage: brain, heart, kidney, vessels	
Search for secondary causes of hypertension	

^a^by CKD-EPI equation

In the 12-lead electrocardiogram (ECG), the findings of left ventricular hypertrophy (LVH), left bundle branch block, and myocardial infarction are regarded as high risk for CVD. Proteinuria or hematuria suggests chronic kidney disease (CKD), and glycosuria suggests diabetes mellitus (DM). Blood hemoglobin and hematocrit levels can determine anemia. An increase in the volume of red blood cells is related to an increase in BP, but its correlation coefficient is very low. If hypokalemia is observed in the baseline evaluation, it suggests excessive state of inorganic corticoids such as primary aldosteronism as a cause of HTN. In addition, baseline potassium levels should be evaluated because thiazide or loop diuretics can lose serum potassium. Hypokalemia is associated with increased lethargy, arrhythmias, and the incidence of diabetes. Conversely, hyperkalemia can be indicated by impaired renal function. An increase in serum creatinine level or a decrease in the calculated glomerular filtration rate (GFR) (< 60 mL/min/1.73 m^2^) indicates a decrease in kidney function. An increase in uric acid level is observed in gout, impaired renal function, obesity, or diuretic use. Estimation of fasting blood glucose and lipid tests are necessary for confirming hyperglycemia and dyslipidemia, respectively. In addition, when diuretics and beta-blockers are used over the long term, the incidence of hyperglycemia and dyslipidemia increases, which can be evaluated by routine tests. Increased cardiac/thoracic ratio on chest X-ray shows cardiac hypertrophy, an increase pulmonary perfusion and pulmonary edema suggests heart failure. Calcification of the aortic arch shows arteriosclerosis. Basic laboratory tests should be evaluated at the first visit and annually.

#### Cardiovascular risk factors and subclinical organ damage

HTN is frequently accompanied by other CV risk factors, therefore BP reduction alone is insufficient to control the overall CV risk [21]. In patients with high CV risk estimated or with target organ damage, lifestyle modification for BP reduction is recommended even in the prehypertensive range. However, there is little data for CV risk stratification for Korean patients with HTN. Table 5 shows the factors in addition to the BP level that are used for evaluation of the risk for future CV events, such as 1) risk factors: age, smoking, obesity, dyslipidemia, increased fasting blood glucose, familial history of premature CVD, and DM; 2) signs of subclinical organ damage: (micro) albuminuria, LVH, retinopathy, atherosclerosis, and increased arterial stiffness; 3) clinical CVD: cerebrovascular disease, heart disease, CKD, and peripheral vascular disease [22]. These predictors of the individual patient’s risk are very useful for making clinical decisions, and studies are therefore needed to develop a risk-stratification system specific to the Korean population.

**Table 5:** Cardiovascular risk factors and subclinical organ damage

Risk factors for cardiovascular disease	
• Age (men ≥45 years old, female ≥55 years old)^a^	
• Smoking	
• Obesity (body mass index ≥25 kg/m^2^) or abdominal obesity (waist circumference men > 90 cm, women > 85 cm)	
• Dyslipidemia [total cholesterol ≥220 mg/dL, low-density lipoprotein (LDL)-cholesterol ≥150 mg/dL, high-density lipoprotein (HDL)-cholesterol < 40 mg/dL, triglycerides ≥200 mg/dL]	
• Pre-diabetes [impaired fasting glucose (100 ≤ fasting blood glucose < 126 mg/dL) or impaired glucose tolerance]	
• Family history of premature cardiovascular disease (men < 55 years, women < 65 years)	
• Diabetes mellitus [fasting blood glucose ≥126 mg/dL, postprandial 2-h glucose (oral glucose tolerance test) ≥200 mg/dL, or hemoglobin A1C ≥6.5%]	
Subclinical organ damage	
• Brain – periventricular white matter hyperintensity (PWMH), microbleeds, asymptomatic cerebral infarction	
• Heart – left ventricular hypertrophy, angina pectoris, myocardial infarction, heart failure,	
• Kidney – albuminuria, decreased estimated glomerular filtration rate (eGFR) (eGFR < 60 ml/min/1.73m^2^, chronic kidney disease)	
• Blood vessels – atherosclerotic plaque, carotid-femoral pulse wave velocity > 10 m/sec, brachial-ankle pulse wave velocity > 18 m/sec, coronary calcification.	
• Retina - stage 3 or 4 hypertensive retinopathy	
Clinical cardiovascular or renal diseases	
• Brain – Stroke, transient ischemic attack, vascular dementia	
• Heart – angina, myocardial infarction, heart failure, atrial fibrillation	
• Kidney – chronic kidney disease stage 3, 4, or 5.	
• Blood vessels – aortic aneurysm, aortic dissection, peripheral vascular diseases	

^a^Age ≥ 65 regarded as 2 risk factors

#### Risk stratification system of hypertension

The risk stratification of HTN in Korea was based on the Korean Medical Insurance Corporation (KMIC) study data, which were drawn from a population with the following characteristics: 1) individuals registered in the early 1990s; 2) relatively young age range of 35–59; and 3) relatively high socio-economic status [23]. As a result, this stratification may have limitations and may underestimate the absolute CV risk of HTN of the general population. Therefore, based on the KMIC data, this guideline has defined the average risk as a 2-fold higher risk than ‘the lowest risk group’, and categorizes the overall risk into three groups: ‘the lowest risk group’, ‘the moderate added risk group’ as higher risk than the average risk, and ‘the high added risk group’ as a 2-fold higher risk than the moderate risk group [24, 25]. The lowest CV risk was identified the patients in their 40s with HTN in the KMIC data with a 2–3% (about 2.5%) 10-year CV risk, corresponding to a 10-year CV event rate for ‘the average risk’ patients of 5%. Accordingly, the 10-year CV event rates for the lowest, the low added, the moderate added, and the high added (including the highest added risk group) risk groups were < 5%, 5–10%, 10–15%, and ≥ 15% (≥20% for the highest risk group), respectively. These levels correspond to the rates of < 5%, 5–15%, 15–20%, and ≥ 20% in the European guideline [1].

In the risk table derived from the KMIC data, patients with the grade 1 HTN who are in their 40s and have no other CV risk factors have a risk of 4.3–5.3%; some of them may exceed the average risk, whereas the women in this group have a risk of 4.0–4.9%, which is below the average risk [23]. Because the KMIC risk table categorized age as 10-year units, it limits the precise changes in CV risk with respect to age and sex. However, women with HTN over age 50 have a clearly higher CV risk than the average. Therefore, ageing ≥45 years in men and ≥ 55 years in women are considered CV risk factors. If the patients aged over 50 years have a systolic BP ≥130 mmHg, smoking and hypercholesterolemia, their 10-year CV risk is estimated to be 9.8–11%, meaning that even a patient with only prehypertension could be stratified as at least moderate added risk if the individual has 3 risk factors. Subjects in their 60s with prehypertension and 3 risk factors clearly belong to the high added-risk group.

Compared with the CV risk score derived from the Korean Heart Study (KHS), the atherosclerotic CVD (ASCVD) risk score (white) of the American Heart Association/American College of Cardiology overestimates CVD risk of the Korean population by 20–60% especially for high risk patients. The CV risk score derived from the KHS is well correlated with that of the KMIC data, and better estimates the risk score in the elderly population because the mean age of KHS is older than the KMIC [26]. As a result of age-based simulation, most individuals aged 65 years or older are classified as high risk individuals from the pre-hypertensive stage, thus this guideline doubles the age risk if the age is over 65 years old [27–29]. However, it is limitation that there is no direct evidence from epidemiology data derived from a national-representative population. Therefore, better-designed prospective observational studies are needed to provide a more representative or clearer estimation of individual risk. The CV risk can be stratified using the BP level, number of risk factors, evidence of subclinical organ damage, and clinical CVDs, as shown in Table 6.

**Table 6:** Stratification of global cardiovascular event for hypertension patients. BP, blood pressure; DM, diabetes mellitus

BP (mmHg)	Prehypertension (130–139/80–89)	Hypertension I (140–159/90–99)	Hypertension II (≥160/100)
Risk			
Risk factor 0	Lowest risk group	Low added risk group	Moderate to high added risk group
Risk factor 1–2	Low to moderate added risk group	Moderate added risk group	High added risk group
Risk factor ≥ 3, DM, sub-clinical organ damage	Moderate to high added risk group	High added risk group	High added risk group
DM^a^, cardiovascular disease, chronic kidney disease	High added risk group	High added risk group	High added risk group

^a^Complicated by sub-clinical organ damage or cardiovascular diseases

#### Symptoms of and screening tools for secondary hypertension

The prevalence of secondary HTN is approximately 5% of all patients with HTN. Additional testing should be performed in the following cases: 1) secondary HTN suggested by age, medical history, physical examination, basic laboratory examination, and the severity of HTN; 2) poor response to antihypertensive drugs; 3) BP resistant to previously effective treatment for no apparent reason; and 4) sudden onset of HTN. In some cases, secondary HTN can be cured by surgery or drug therapy. Renovascular HTN is suspected among patients with HTN beginning at the age of ≤30 or ≥ 55 years, worsening of previously well-controlled HTN, an abdominal bruit, resistant HTN, an increase in the creatinine level of > 30% over the baseline level following the administration of an angiotensin converting enzyme (ACE) inhibitor or angiotensin II receptor blocker (ARB), and the presence of atherosclerotic disease in other organs. Screening for renovascular HTN is performed by using the captopril renal scan, Doppler ultrasound (US), computed tomography (CT), or magnetic resonance angiography. Hypokalemia with no apparent cause or an incidentally diagnosed adrenal mass are indications for evaluation for hyperaldosteronism. Paroxysmal and/or refractory HTN accompanied by hyperadrenergic symptoms suggests the possibility of pheochromocytoma, which is an indication for measurement of the catecholamine level in the plasma and/or 24-h urine, CT, magnetic resonance imaging, or radioisotope imaging (I-131 metaiodobenzylguanidine) (Table 7). In recent years, sleep apnea syndrome has been suggested as the leading cause of secondary HTN. Although there is still no evidence of the benefits for or the confirmation of screening tests, the prevalence of sleep apnea is high among obese patients or those with resistant HTN and can be screened by self-questionnaire or diagnosed by sleep polysomnography.

**Table 7:** Clinical clues and diagnoses of secondary hypertension

Diseases	Clinical clues		Diagnoses		
	History	Physical diagnosis	Chemistry	Screening test	Additional test
Parenchymal renal diseases	Urinary tract infection or obstruction, analgesic abuse, familial history of polycystic kidney disease	Abdominal mass (polycystic kidney disease)	Proteinuria, hematuria, pyuria, reduced glomerular filtration rate (GFR)	Renal ultrasound (US)	Further studies for kidney diseases
Renal artery stenosis	Fibromuscular dysplasia, premature hypertension (female), atherosclerotic diseases, sudden onset or worsening of hypertension, resistant hypertension, recurrent pulmonary edema	Abdominal bruit	Rapid worsening of renal function [spontaneous or after angiotensin converting enzyme (ACE) inhibitor or angiotensin II receptor blocker (ARB) treatment]	Kidney size difference > 1.5 cm, Duplex Doppler US, computed tomography (CT)	Magnetic resonance imaging, digital subtraction angiography
Primary aldosteronism	Muscle weakness, premature hypertension, familial history of premature stroke (< 40 years)	Arrhythmia (severe hypokalemia)	Hypokalemia (spontaneously or after treatment by diuretics), incidental adrenal mass	Aldosterone-renin ratio (after correction of hypokalemia and excluding effect of ACE inhibitor or ARB)	Suppression test by saline infusion, fludrocortisone, and/or captopril); adrenal CT, adrenal vein sampling
Pheochromo-cytoma	Paroxysmal hypertension, emergency visit by persistent hypertension with headache, sweat, and/or pallor, familial history	Café-au-lait lesion and neurofibromatosis neurofibroma	Incidental adrenal mass (extraadrenal mass in some cases)	Metanephrine and/or nor-metanephrine in 24-h urine	Abdominal and/or pelvic CT or magnetic resonance imaging (MRI); radioisotope scan using meta-iodobenzyl-guanidine
Cushing syndrome	Rapid weight gain, polyuria, polydipsia, psychiatric problems	Central obesity, moon face, buffalo hump, abdominal striae, hirsutism	Hyperglycemia	Cortisol in 24-h urine	Dexamethasone suppression test

## Chapter II. Treatment of hypertension

### Treatment of hypertension

The purpose of HTN treatment is to prevent CVD caused by increased BP and to reduce mortality by controlling high BP. In patients who already have established CVD, treatment aims to control BP to prevent progression or recurrence of disease in order to decrease mortality and improve quality of life. HTN treatment provides greater benefit in patients who are at higher risk for CVD. Most clinical studies of HTN have found that lowering SBP by approximately 10–20 mmHg or DBP by approximately 5–10 mmHg can reduce the occurrence of stroke by 30–40% and that of ischemic heart disease by 15–20% [30]. Because most clinical studies were performed over a relatively short period, the benefits of HTN treatment over a period of 5 years or more seem to be much more pronounced than the treatment efficacy observed in clinical studies. The benefits of HTN treatment are not affected by sex or age and are similar for the treatment of systolic HTN in the elderly. HTN treatment was found to be the most cost-effective intervention for prevention of CVD.

#### Strategy for hypertension treatment

**Fig 1:**
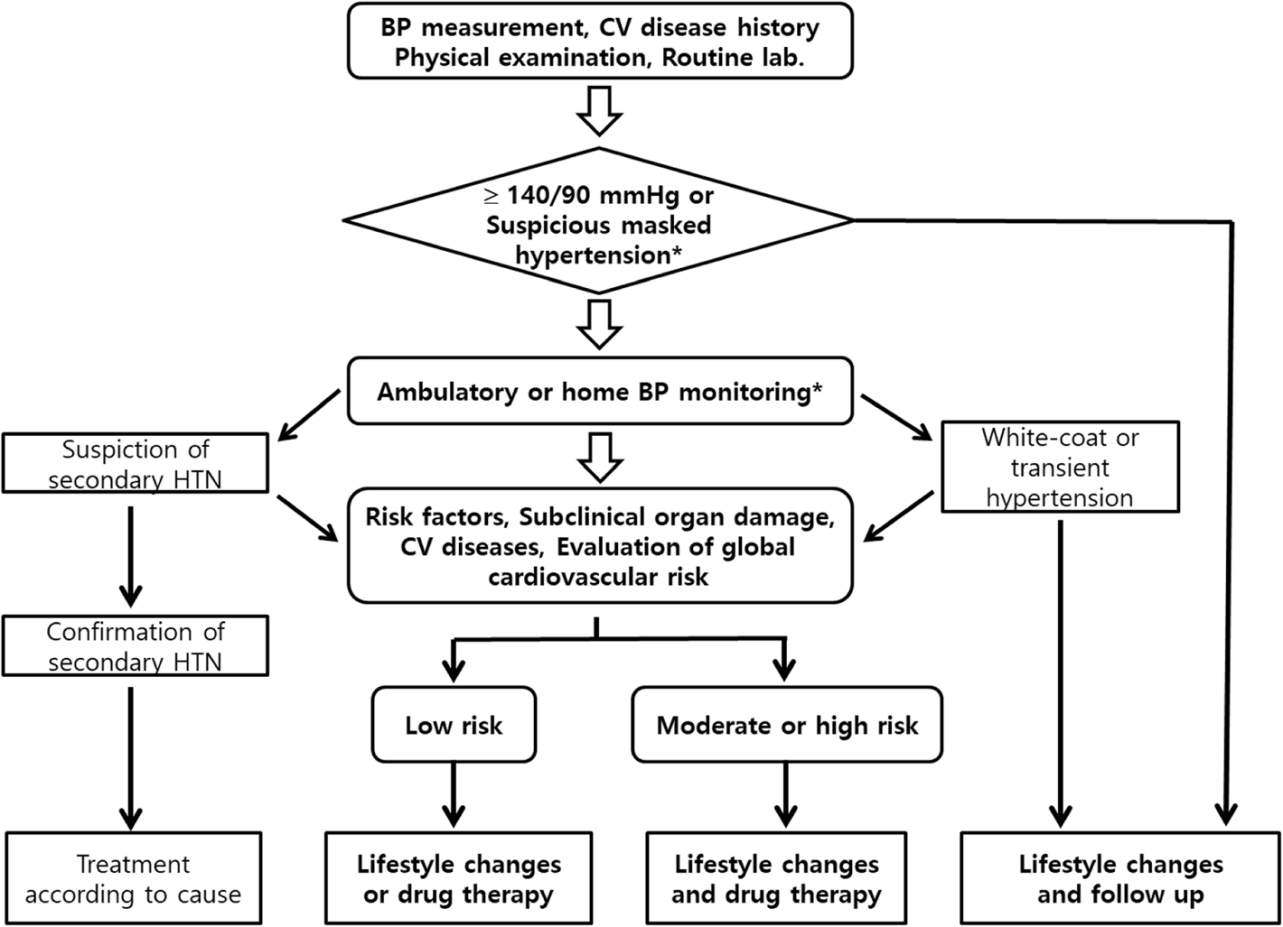
Treatment strategies for hypertension, BP; blood pressure, CV; cardiovascular, HTN; hypertension, *; recommended test

If a patient is already known to have high BP, the diagnosis of HTN must be confirmed prior to treatment by measuring the out-of-clinic BP, such as at home BP or using 24-h BP monitoring. Measurement of out-of-clinic BP helps not only to obtain a more accurate diagnosis but also to determine the appropriate treatment for the patient and to increase treatment adherence. If HTN is diagnosed, the risk factors for CVD, associated diseases, and existence of hypertensive complications should be investigated (Fig. 1).

Patients with white coat HTN, defined as high BP in but not out of the clinic, must be followed up periodically at 3–6-month intervals because their risk of CVD increases over time. HTN treatment must include non-drug therapy (such as lifestyle modifications) concomitant with drug therapy. The initiation of drug therapy needs to be considered and determined on the basis of not only the BP level but also the presence of risk factors for CVD and evidence of damage to target organs. Drug therapy may be used in patients with a BP of 140/90 mmHg or higher regardless of the existence of other risk factors or associated diseases. The quality of life of patients with HTN can be affected by physical and psychological problems caused by HTN, the main and side effects of the drug, and the relationship between the patient and physician. Adequate communication and provision of information can decrease the dosage and frequency of medication used, which increases patient adherence, improves the BP control rate, and promotes continuous treatment.

#### Initiation of hypertension treatment

##### Prehypertension

**Table:** 

**Recommendations**	**Class**	**Level**	**References**
• Generally, drug therapy is not recommended in prehypertension.	**III**	**A**	[31, 32]
• In populations with elevated BP and in the prehypertensive range, instructions for lifestyle modifications should be provided for the prevention of HTN development and CVD.	**I**	**B**	[33–36]
• In the high risk HTN patients* with systolic BP over 130 mmHg by AOBP measurement, it is recommended to provide drug therapy along with lifestyle modifications.	**IIa**	**B**	[37]
• In the population within the prehypertensive range, ambulatory BP monitoring or home BP measurement is recommended to exclude masked HTN.	**IIa**	**B**	[38]

*Patients ≥50 years old with CVD, PAD, aortic disease, heart failure or LVH

CV mortality caused by HTN increases 2-fold for each 20-mmHg increase in SBP or 10-mmHg increase in DBP over the baseline level of 115/75 mmHg. Therefore, in patients with BP over 120/80 mmHg, non-drug therapy is recommended to prevent the occurrence of HTN and CV events [31, 32].

Initiation of drug therapy in patients with prehypertension can delay the progression to HTN [39, 40], but there is little evidence for the effectiveness of early intervention in most clinical studies [32]. The Heart Outcome Prevention Evaluation-3 (HOPE-3) trial in the intermediate-risk group also had no effect on drug therapy, but an additional analysis suggested the possibility of drug treatment in patients with BP over 140 mmHg [31]. Initiation of drug therapy in the prehypertensive range showed no consistent benefit in patients with prediabetes [41, 42], DM [43], stroke [44], or coronary artery disease (CAD) [45]; therefore, the cost-benefit aspect should be considered in the decision to use drug therapy (Table 8).

**Table 8:**
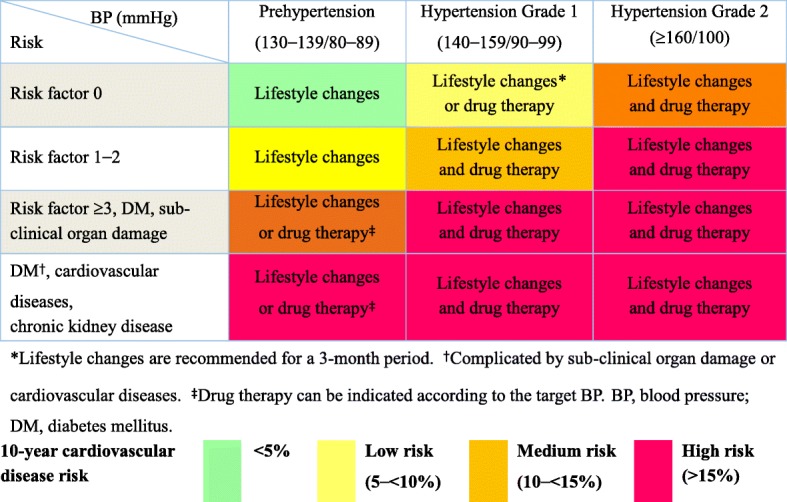
Drug treatment for hypertension according to the risk. BP, blood pressure; DM, diabetes mellitus

Systolic Blood Pressure Intervention Trial (SPRINT) subgroups whose systolic BP are more than 130 mmHg measured by AOBP, who have a history of CAD, peripheral vascular disease, aortic disease, heart failure, or LVH, may consider drug therapy on top of intensive lifestyle modifications [37]. The reason for restricting drug therapy for SPRINT subgroups is that the definition of high risk patients assessed by the ASCVD risk score is not suitable for Korean patients and cannot be generalized for the elderly and patients with CKD.

##### Grade 1 hypertension

**Table:** 

**Recommendations**	**Class**	**Level**	**References**
• In patients with grade 1 HTN at low risk, BP-lowering drug treatment is recommended if the patient remains hypertensive after a period of lifestyle intervention.	**I**	**B**	[31, 46, 47]
• In patients with grade 1 HTN and at moderate-to-high risk, prompt initiation of drug treatment is recommended along with lifestyle interventions.	**I**	**A**	[31, 48, 49]

Patients with grade 1 HTN without any other risk factors are in the low risk group and would not be expected to obtain a greater benefit from treatment [50]. However, their overall risk will increase over time, and the window in which treatment could reverse progression might be missed. In addition, the risk of CVD is low, thus lifestyle modifications can be actively attempted before starting drug treatment. However, it is recommended to begin drug treatment as soon as possible when lifestyle modification is considered ineffective, when CV risk factors are revealed during lifestyle modification, and when patients are unable to regularly visit and receive guidance of lifestyle modification. In general, it is desirable to consider lifestyle modification as an adjunct, not as an alternative to medication. Current antihypertensive drugs are generally inexpensive and safe, and drug therapy has been found to be cost-effective given that patients generally fail to accomplish lifestyle changes. Drug therapy is recommended only after measurement of the out-of-clinic BP in order to exclude the possibility of white coat HTN [51, 52]. The effect of drug therapy on white coat HTN has not yet been demonstrated; however, as white coat HTN poses increased metabolic risk and risk for CV events over the long term, lifestyle modification is recommended at first, and patients should be observed periodically for development of persistent HTN [31, 53]. Drug therapy should be instituted immediately in patients with high risk grade 1 HTN [46, 48, 49].

##### Grade 2 hypertension

**Table:** 

**Recommendations**	**Class**	**Level**	**References**
• In patients with grade 2 HTN, prompt initiation of drug treatment is recommended along with lifestyle interventions.	**I**	**A**	[46, 48, 49]

According to most randomized clinical trials and meta-analyses, immediate drug therapy is warranted in patients with a BP of ≥160/100 mmHg due to the significant benefit of drug treatment [32, 48, 49].

##### Hypertension in the elderly

**Table:** 

**Recommendations**	**Class**	**Level**	**References**
• BP-lowering drug treatment and lifestyle modifications are recommended for fit older patients (> 65 years but not > 80 years) when SBP is over 140 mmHg.	**IIa**	**B**	[54]
• BP-lowering drug treatment and lifestyle modifications are recommended for frail older patients or older patients (> 80 years) when SBP is over 160 mmHg.	**I**	**A**	[54, 55]

The effect of drug therapy against HTN is clear irrespective of age. BP-lowering drug treatment and lifestyle modifications are recommended for frail older patients or older patients (> 80 years) when SBP is over 160 mmHg. Drug therapy can be considered if the SBP is 140–159 mmHg and the patient tolerates hypertensive medications well.

#### Target blood pressure in the treatment of hypertension

**Table:** 

**Recommendations**	**Class**	**Level**	**References**
• For hypertensive patients at low to moderate risk, target BP of 140/90 mmHg is recommended.	**I**	**A**	[31, 53]
• For patients with CVD (over age 50 with CAD, PAD, aortic disease), congestive heart failure (CHF), or LVH, a target BP of 130/80 mmHg can be considered (SPRINT eligible population).	**IIa**	**B**	[37]
• For high risk patients with 10-year CVD risk of > 15%, a target BP of 130/80 mmHg can be considered*.	**IIa**	**B**	[56, 57]

*Elderly (≥65 years old), CKD patients will be recommended separately.

**Fig. 2:**
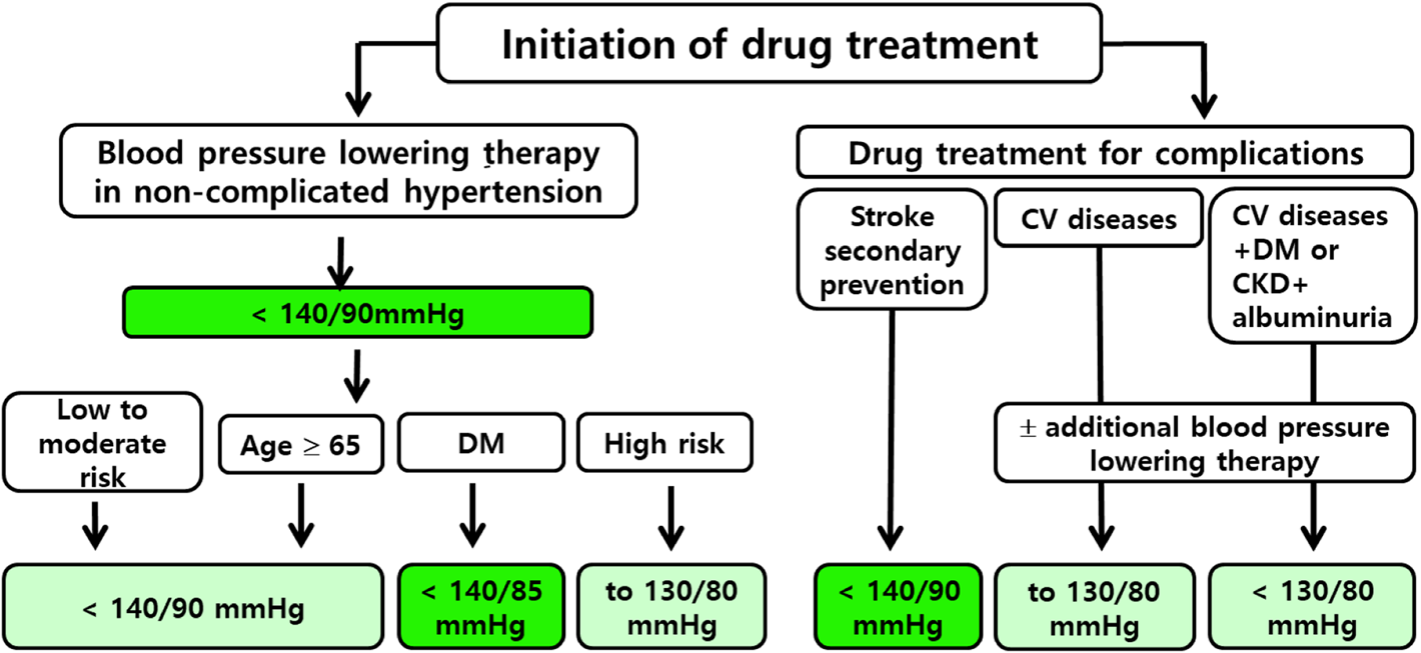
The algorithm and level of evidence of target BP in various clinical conditions

Except under specific circumstances as shown in Table 9, the target BP is generally a SBP of less than 140 mmHg and DBP of less than 90 mmHg [32, 58, 59]. For patients with CVD (over age 50 with CAD, PAD, aortic disease), CHF, or LVH, a target BP of 130/80 mmHg can be considered (SPRINT eligible population) [37, 56]. In contrast, for hypertensive patients at low-to-moderate risk, a target BP of 140/90 mmHg is recommended [31] (Fig. 2).

**Table 9:** Summary of office blood pressure target goal. BP, blood pressure; DM, diabetes mellitus

Conditions	Systolic BP (mmHg)	Diastolic BP (mmHg)
Uncomplicated, general	< 140	< 90
Elderly	< 140	< 90
DM		
Uncomplicated	< 140	< 85
Complicated^a^	< 130	< 80
High risk^b^	≤ 130	≤ 80
Cardiovascular disease	≤ 130	≤ 80
Cerebrovascular disease	< 140	< 90
Chronic kidney disease		
No albuminuria	< 140	< 90
Albuminuria^c^	< 130	< 80

^a^Complicated by sub-clinical organ damage or cardiovascular diseases. ^b^High risk, elderly patients should be followed elderly patients criteria. ^c^including microalbuminuria

##### Hypertension in the elderly

**Table:** 

**Recommendations**	**Class**	**Level**	**References**
• For elderly hypertensive patients, a target SBP < 140 mmHg can be considered.	**IIa**	**B**	[54, 60, 61]

Although it has previously been reported that there was no difference in prognosis between BP targets of 140 mmHg and 150 mmHg [62], the SPRINT trial showed clear evidence of BP lowering effects in elderly patients with HTN [37]. Therefore, the target SBP is below 140 mmHg with a DBP that is not excessively low, i.e., less than approximately 60 mmHg [62, 63] (Fig. 2).

##### Hypertension in patients with diabetes mellitus

**Table:** 

**Recommendations**	**Class**	**Level**	**References**
• It is recommended that SBP be lowered to below 140 mmHg in hypertensive patients with diabetes.	**I**	**A**	[59, 64–67]
• It is recommended that DBP be lowered to below 85 mmHg in hypertensive patients with diabetes.	**I**	**B**	[68, 69]
• In diabetic patients with CVD, a target BP < 130/80 mmHg can be considered.	**IIa**	**C**	[70]

Reduction of BP in patients with HTN and DM is very important for reducing CV complications [71]. Few studies have shown reduction of SBP to below 130 mmHg. Even reduction of SBP to less than 120 mmHg did not demonstrate any additional preventive effect on CVD but rather showed a deleterious effect on renal function; therefore, the recommended target BP is an SBP below 140 mmHg [59, 64–67] and a DBP below 85 mmHg [68, 69].

The Action to Control Cardiovascular Risk in Diabetes (ACCORD) study sub-analysis of a SPRINT-eligible population showed an overall reduction in CV events with intensive BP lowering to < 130/80 mmHg, therefore in DM patients with CVD, a target BP < 130/80 mmHg can be considered [70] (Fig. 2).

##### Hypertension in patients with stroke

HTN is a most important causative risk factor for stroke. HTN treatment can reduce the recurrence of stroke and CV events [72–74], but there are no distinct benefits from reducing SBP to below 130 mmHg [75]. In particular, a recent clinical study in patients with cerebral infarction showed no additional benefit from controlling SBP below 140 mmHg [76]. Considering the clinical studies to date, a target SBP below 140 mmHg is recommended in patients with stroke (Fig. 2).

##### Hypertension in patients with coronary artery disease

Reduction of SBP to below 130 mmHg shows no consistent prevention of CVD in patients with HTN and CAD [77–79]. However, based on multiple meta-analyses and SPRINT study result, a target SBP of around 130 mmHg can be considered [56, 57, 77] (Fig. 2).

##### Hypertension in patients with chronic kidney disease

**Table:** 

**Recommendations**	**Class**	**Level**	**References**
• For CKD patients with HTN, target BP of 140/90 mmHg is recommended.	**I**	**A**	[64, 80, 81]
• For CKD patients with HTN and albuminuria, a target BP <130/80 mmHg can be considered.	**IIa**	**B**	[22, 82]

The main aim of controlling BP in patients with CKD is to prevent deterioration of renal function and reduce the occurrence of CVD. Further control of SBP to below 140 mmHg has shown no additional benefit in patients with HTN and CKD without DM [83–85]. However, the data on the goal of treatment in patients with HTN and CKD with DM is even scantier [82, 86]. Meta-analyses have not proven that a target BP of less than 140 mmHg is any more effective at preventing cardiac and renal events [64, 80, 81]. Therefore, based on recent clinical data, a target SBP of less than 140 mmHg is recommended regardless of the presence of DM. However, a target SBP below 130 mmHg can be recommended in patients with HTN with prominent albuminuria [22] (Fig. 2). Even though the SPRINT study enrolled CKD patients, the results cannot be generalized to recommend a target SBP < 130 mmHg overall for CKD patients [87].

##### Lower limit blood pressure of hypertension treatment

**Table:** 

**Recommendations**	**Class**	**Level**	**References**
• If SBP drops to 110 mmHg and DBP falls to below 70 mmHg, the risk of mortality and the risk of developing CAD may increase. Lowering DBP to below 70 mmHg should be carefully considered in the elderly, in DM, and in multiple CAD without revascularization, and HTN patients with LVH.	**IIb**	**C**	[88–91]

As BP increases, the CV risk also increases, whereas as BP decreases, the risk of the occurrence of a CV event decreases to some extent. There is insufficient clinical data to prove the J-curve hypothesis (the hypothesis that excessive lowering of SBP and DBP will increase rather than decrease CV events and mortality), however a post-hoc analysis of clinical studies suggests the possibility of such a J-curve effect and a pathophysiological detriment of excessively low BP [92]. Therefore, it is not recommended to target BP too low, but additional studies are needed to determine how low a BP is desirable. That is, 1) elderly people who are likely to be associated with CAD; 2) patients with multiple CAD who are not undergoing revascularization; 3) DM patients who are likely to be associated with multiple CAD; 4) In HTN patients with LVH, it can be considered not to lower DBP below 70 mmHg [88–91].

##### Target blood pressure according to blood pressure measurement methods

According to the measurement method, the BP outside the clinic is lower than the office BP by about 5 mmHg for both SBP and DBP on average. The target BP is 140/90 mmHg measured by office BP may correspond to a mean home BP, the day-time BP or 24-h ambulatory BP monitoring of 135/85 mmHg, 135/85 mmHg, and 130/80 mmHg, respectively. However, since the difference in BP measured by the different BP measurement method is largely uneven depending on the level of the BP and the individual characteristics, and is not constant at the time of measurement, it is necessary to consider the individual differences when converting the target BP [93]. In particular, target BP according to an AOBP measurement may overlook the masking effects.

### Non-pharmacologic therapy and lifestyle modifications

**Table:** 

**Recommendations**	**Class**	**Level**	**References**
• Lifestyle modification is recommended in the population with elevated BP and prehypertension and all hypertensive patients.	**I**	**A**	[94–97]

Non-pharmacologic therapy or lifestyle modifications, such as adoption of a healthy diet, exercise, smoking cessation, and moderation of alcohol intake, has shown great impact to lower BP and is strongly recommended in all patients with HTN and also in populations with elevated BP and within the prehypertensive range (Table 10). Healthy lifestyle habits have almost an equipotent BP-lowering effect as approximately one dose of antihypertensive drug [98]. Furthermore, in patients with HTN who are using medication, adding lifestyle modification can reduce the dose and frequency of medication used, maximize the effects of the drug, and reduce side effects. Lifestyle improvement also has other beneficial effects on CV risk in addition to the lowering of BP. However, clinicians should remain aware that it is difficult to maintain lifestyle modifications long-term or to achieve a target BP for the HTN in grade 2 or higher even with the best attempts. Therefore, the clinician should provide encouragement to continue lifestyle modifications while also educating the patient in their limitations. In addition, because adopting several types of lifestyle modification rather than one alone maximizes the effects, a simultaneous approach is recommended to meet the goal of minimizing CVD.

**Table 10:** Blood pressure reduction by lifestyle modification

Lifestyle modification	BP reduction (systolic/diastolic BP, mm Hg)	Recommendation
Restriction of salt intake	-5.1/-2.7	Less than 6 g of salt per day
Body weight reduction	-1.1/-0.9	Each reduction of 1 kg
Moderation in drink	-3.9/-2.4	Less than two glasses per day
Exercise	-4.9/-3.7	30–50 min per day for more than 5 days in a week
Diet control	-11.4/-5.5	Vegetables-based healthy diet habit*

*Diet rich in vegetables, fruits, and fish and low in fat and calorie

#### Restriction of salt intake

**Table:** 

**Recommendations**	**Class**	**Level**	**References**
• Salt restriction to < 6 g per day is recommended.	**I**	**A**	[94–96, 99–101]

It is estimated that Koreans consume 10 g of salt daily (sodium 3.9 g), which is a higher amount than Western recommendations (salt 5 g). The main source of salt in Korea is spices, vegetables, and grains [102–104].

Salt intake can increase the risk for CV events because of the association of central hemodynamics rather than on peripheral BP [105]. Halving the daily salt intake of 10 g will decrease SBP by 4–6 mmHg [106, 107]. However, there have been variable and confusing reports about the relationship between salt restriction and CV events [99–101, 108]. Despite the absence of randomized large-scale trials, some reports of a J-curve phenomenon for salt intake and CV events, and lack of Korean data, there is no clear evidence that salt restriction is harmful, especially among Koreans with high salt intake. Therefore, we must recommend decreasing salt intake in accordance with other guidelines [109].

The daily recommended amount of salt is less than 6 g [sodium (g) × 2.5]. Salt reduction has many benefits, including lowering of BP and reducing the need for diuretics, which cause detrimental urinary loss of potassium and calcium. The avoidance of urinary calcium loss prevents the development of osteoporosis and renal calculi.

Sensitivity to salt tends to be higher in patients who are elderly, obese, or have diabetes or family members with HTN. Greater salt sensitivity means a greater reduction in BP in response to salt restriction. Because of the high correlation with salt intake and energy intake, reducing caloric intake can reduce salt intake. Dietary habits must be modified. Some recommendations are not to put additional salt on the table during the meal and to avoid high-salt processed foodstuffs. Some frequently eaten foods, such as kimchi, stew, soup, salted fermented seafood, instant ramen, and dry bar snacks containing meat and fish, are very salty. When cooking, natural ingredients should be used instead of synthetic flavoring agents. The patient should reduce absolute salt intake but also try to consume more low-salt foods.

Eating potassium-rich foods can help prevent HTN, and can lower BP in hypertensive patients. Potassium can inhibit BP rise with salt intake by excreting sodium outside the body. The higher the potassium intake, the more potent the BP-lowering effect of potassium can be achieved. However, attention should be paid to potassium intake in patients with impaired renal function.

#### Weight reduction

**Table:** 

**Recommendations**	**Class**	**Level**	**References**
• Body weight control to reduce BMI < 25 kg/m^2^ is recommended for BP reduction.	**I**	**A**	[97, 110]

HTN is closely associated with obesity, and weight reduction decreases BP. Central obesity in particular is closely associated with HTN, dyslipidemia, diabetes, and CV death. In a patient heavier than 110% of the ideal body weight, a weight reduction of only 5 kg can decrease BP. The beneficial effects of weight reduction are higher in patients with diabetes, dyslipidemia, and LVH. The combination of weight reduction with exercise, moderation of alcohol consumption, and reduction of salt intake has synergistic effects on BP. The recommended initial goal of weight reduction is 4–5 kg, with an additional 5 kg reduction after the initial goal has been achieved.

The ideal BMI [weight (kg)/height (m^2^)] varies among reports and according to nationality. A collaborative analysis reported that BMI either above or below the apparent optimum of approximately 22.5–25 kg/m^2^ is itself a strong predictor of overall mortality [110]. Another meta-analysis found that both overweight and grade 1 obesity were associated with significantly lower all-cause mortality [110]. A report on data from 1.2 million Koreans revealed that the risk of death from any cause was lowest among patients with a BMI of 23.0 to 24.9 kg/m^2^ and recommended a BMI of less than 25 kg/m2. Unfortunately, there are no Korean-specific data on appropriate waist circumference. A waist circumference of less than 90 cm for men and 85 cm for women is recommended for Asian individuals.

The recommendations for weight reduction are to eat breakfast every morning, eat slowly, and avoid a high-carbohydrate diet, alcohol, snacks such as bread and cookies, and sweetened beverages. A high-fiber diet is recommended and a high-fat diet including food fried with oil is prohibited. Patients should try to eat as many fruits and vegetables as possible and to avoid meals containing large amounts of cholesterol and saturated fatty acids.

#### Moderation of alcohol consumption

**Table:** 

**Recommendations**	**Class**	**Level**	**References**
• It is recommended to moderate alcohol consumption to less than 2 drinks per day.	**I**	**A**	[111]

BP tends to increase in patients who drink excessive amounts of alcohol and such patients are also resistant to antihypertensive drugs. An appropriate moderate daily amount of alcohol is less than 20–30 g for men or 10–20 g for women. A man or woman with lower-than-average body weight is more sensitive to alcohol and is therefore permitted half of the recommended amount. Heavy drinkers should be warned that they are high risk for stroke. One bottle of beer (720 mL), 1 glass of wine (200–300 mL), 1 glass of sake (200 mL), 2 shots of whisky (60 mL), or 2–3 glasses of soju corresponds to 30 g of alcohol.

#### Exercise

**Table:** 

**Recommendations**	**Class**	**Level**	**References**
• Regular aerobic exercise (e.g. at least 30 min of moderate dynamic exercise 5–7 days per week) is recommended.	**I**	**A**	[22, 112]
• It is recommended that isometric exercise or isometric exercise, such as lifting a heavy weight, can be performed concurrently with aerobic exercise, but should be avoided as BP may temporarily rise when BP is not controlled.	**I**	**A**	[22, 112]

The benefits of regular exercise are lowering of BP, improvement of cardiopulmonary function, reduction of body weight, improvement of the lipid profile (including elevation of HDL-cholesterol), and reduction of emotional stress. Aerobic exercises such as brisk walking, jogging, bicycling, swimming, jumping rope, playing tennis, and aerobic dancing are recommended for patients with HTN [113]. The appropriate intensity of exercise is 60–80% of the maximal heart rate (220 minus age in years). Such exercise should be performed 5–7 times per week. Aerobic exercise should begin at low intensity for 10–20 min and then increase to appropriate intensity for another 30–60 min. It is best to exercise for 90–150 min or more per week. Warm-up and finish exercises before and after exercise session for five minutes is necessary. In addition, isotonic muscle strength and isometric exercise using muscular dumbbells two or three times a week is recommended not only to reduce BP but also to improve metabolic factors and strengthen muscles [114]. Isometric exercises using a hand dynamometer is used to estimate the maximal handgrip power and then is held for 2 min at a strength of 30–40% of the maximum measured weight, followed by rest for 1 min for 4 times, three days a week. It is recommended that isometric exercise or isometric exercise, such as lifting a heavy weight, can be performed concurrently with aerobic exercise but should be avoided as BP may temporarily rise when BP is not controlled. Most patients with uncomplicated HTN can begin regular exercise without an initial evaluation and increase the duration and intensity to appropriate levels as possible. However, patients with known CVD or other risk factors are recommended to start the exercise only after complete evaluation by an exercise consultant and to follow a program.

#### Smoking cessation

**Table:** 

**Recommendations**	**Class**	**Level**	**References**
• Smoking cessation, supportive care, and referral to smoking cessation programs are recommended.	**I**	**A**	[115–117]

During smoking, the BP increases temporarily in response to nicotine. Among patients with white coat HTN, smokers maintain a higher daytime ambulatory SBP than do non-smokers with a similar office BP [118]. Because smoking, like HTN, is a powerful risk factor for CVD [115], CV events are inevitable in patients who continue smoking regardless of BP control. Second-hand smoking is also harmful. Smoking cessation should be advised at every opportunity. Low-nicotine-containing replacement materials do not increase BP and can be recommended in combination with behavior therapy. During smoking cessation, regular exercise and diet therapy should be emphasized in order to prevent weight gain.

#### Healthy diet

**Table:** 

**Recommendations**	**Class**	**Level**	**References**
• Increased consumption of vegetables, fresh fruits, fish, nuts, and unsaturated fatty acids; low consumption of red meat; and consumption of low-fat dairy products	**I**	**A**	[82, 119, 120]

BP is lower in vegetarians than in people who mainly eat meat, and maintaining a vegetarian diet can reduce BP. The BP-lowering effect results not from decreasing the intake of animal protein but from increasing the intake of vegetables and fruits in combination with decreasing the intake of saturated fatty acids. In a study in elderly people, BP decreased by 3/1 mmHg when intake of vegetables and fruits was increased alone but by 6/3 mmHg when it was combined with a decrease in fat intake [121–123]. It is important for HTN patients to change the overall dietary pattern rather than diet that emphasizes specific nutrients [119]. For example, Dietary Approaches to Stop Hypertension (DASH), which includes consumption of more fruits, vegetables, and fish and consumes less fat, can lower BP by 11/6 mmHg. Because it contains multiple food groups, the DASH diet is likely to have additional beneficial effects [121, 123, 124]. Regular intake of fish reduces BP in obese HTN patients and improves lipid metabolism. In Korea, diets consisting of tofu, soybean, fruit, vegetables, and fish were associated with a low prevalence of HTN with a high dietary intake [125]. Therefore, a healthy balanced diet such as DASH or a Mediterranean diet for HTN patients is recommended not only to lower BP but also to prevent CVD [119, 120].

#### Others

Caffeine from various foods rapidly increases BP, but the effect does not progress to HTN because tolerance to caffeine develops. Emotional stress increases both BP and the risk for CVD, making the control of emotional stress important for the management and patient adherence of HTN. Further studies are required to examine the long-term effects of stress control on HTN and CVD. The effectiveness of various methods of stress management, such as relaxation and biofeedback, for the management of HTN remains uncertain. There is still no clear evidence for the effects of micronutrients, calcium, magnesium, and supplementary fiber on BP.

### Pharmacological therapy for hypertension

**Table:** 

**Recommendations**	**Class**	**Level**	**References**
• Prompt initiation of BP-lowering drug treatment is recommended in patients with high risk or grade 2 HTN, simultaneous with the initiation of lifestyle for achieving target goal BP.	**I**	**A**	[126–128]

The occurrence of CV events in patients with HTN can be decreased by reducing the BP. Currently available antihypertensive drugs are more effective than placebo for prevention of CVD. This preventive effect is relatively larger for stroke than for CAD. The extent to which CV events are reduced depends on the degree of BP reduction. All major classes of antihypertensive drugs, including beta-blockers and diuretics, are suitable for first-line treatment. However, the individual drug should be prescribed with consideration of the patient’s individual situation, including age, comorbidities, and possible adverse effects. Simplifying the medication schedule, careful monitoring of the adverse effects, and checking the BP at home are useful for improving patient adherence and making the patient an active participant in the treatment.

#### Strategies for prescription of antihypertensive drugs

##### Principles of drug selection

**Table:** 

**Recommendations**	**Class**	**Level**	**References**
• In patients with BP higher than 160/100 mmHg or more than 20/10 mmHg above the target BP, two drugs can be prescribed in combination to maximize the antihypertensive effect and to achieve rapid BP control.	**IIa**	**C**	[129]

For reducing long-term CV morbidity and mortality, it is essential to control most of the modifiable risk factors and to reduce the BP to less than 140/90 mmHg [22]. Drug therapy is initiated at a low dose to avoid adverse effects. The preferred drugs are long-acting and can be taken only once a day [130]. Drugs with a high trough/peak ratio (T/P ratio > 0.5) are helpful for improving adherence and to maintain a stable BP with minimal variability [131]. When it is difficult to control BP with once-daily dosing, a twice-daily schedule is an alternative option. ACE inhibitors, ARBs, calcium channel blockers, beta-blockers, and diuretics are all agents suitable for initiation of antihypertensive treatment. The indications, contraindications, comorbidities, and presence of asymptomatic organ damage should all be considered in the choice of drug. Beta-blockers in the elderly are controversial for treatment benefits and should only be used if there is a specific indication. Concomitant use of beta-blockers and diuretics increases the risk of developing diabetes, so care should be taken in patients at high risk of developing DM [22]. In patients with BP higher than 160/100 mmHg or more than 20/10 mmHg above the target BP, two drugs can be prescribed in combination to maximize the antihypertensive effect and achieve rapid BP control [22]. Single pill combination drugs have multiple benefits, including maximizing reduction of BP, minimizing adverse effects, increasing adherence, and preventing CVD and target organ damage [132].

##### Selection of drugs

It is reasonable to choose drugs according to the patient’s comorbidities and clinical characteristics rather than his or her BP level. There are five available classes of first-line drugs with proven BP-lowering effects, safety, and acceptable adverse effects according to multiple studies. They are: 1) ACE inhibitors or ARBs; 2) beta-blockers; 3) calcium channel blockers; and 4) diuretics such as hydrochlorothiazide, chlorthalidone, or indapamide. All reduce BP to a similar extent when the dose has been adjusted. However, there might be individual differences in BP lowering, adverse effects, and long-term CV events, making it very important to choose the appropriate drugs according to the patient’s combined risk factors and comorbidities (Table 11). No antihypertensive drug is inherently superior, and the drugs most appropriate for the individual patient should be preferred (Table 12).

**Table 11:** Compelling indications for choosing the antihypertensive drugs

Disease conditions	ACE inhibitors or Angiotensin receptor blockers	Beta-blockers	Calcium channel blockers	Diuretics
Congestive heart failure	○	○		○
Left ventricular hypertrophy	○		○	
Coronary artery disease	○	○	○	
Chronic kidney disease	○			
Stroke	○		○	○
Elderly, isolated systolic hypertension	○		○	○
Post-myocardial infarction	○	○		
Prevention of atrial fibrillation	○			
Diabetes mellitus	**○**	○	○	○

**Table 12:** Indications and contraindications of antihypertensive drugs

	Absolute indications	Relative indications	Need cautions	Absolute contraindications
Angiotensin-converting enzyme inhibitors/angiotensin receptor blockers	Congestive heart failure, diabetic nephropathy		(Bilateral) Renal artery stenosis, hyperkalemia	Pregnancy, angioedema
Beta-blockers	Ischemic heart disease, myocardial infarction	Tachyarrhythmia	High blood glucose, peripheral artery disease	Asthma, severe and symptomatic bradyarrhythmia
Calcium channel blockers	Elderly hypertension, isolate systolic hypertension, ischemic heart disease (non-DHP*)		Congestive heart failure	Severe and symptomatic bradyarrhythmia (non-DHP*)
Diuretics	Congestive heart failure, isolate systolic hypertension		High blood glucose	Gout, hypokalemia

*Non-DHP: non-dihydropyridine calcium channel blockers.

#### Classes of antihypertensive drugs

##### Diuretics

**Table:** 

**Recommendations**	**Class**	**Level**	**References**
• Thiazide or thiazide-like diuretics can be used as first-line drugs with a preference for chlorthalidone or indapamide.	**IIa**	**B**	[133, 134]
• Loop diuretics can be considered in patients with CHF, advanced CKD of stage IV or stage V.	**IIa**	**B**	[135]
• In patients with resistant HTN, aldosterone antagonists such as spironolactone can be considered in the absence of hyperkalemia.	**IIa**	**B**	[136, 137]

Diuretics decrease BP initially by reducing reabsorption of sodium in the renal distal convoluted tubules and later by decreasing peripheral vascular resistance. High-dose thiazide-derivative diuretics can induce hypokalemia, glucose intolerance, hyperuricemia, arrhythmia, and adverse lipid metabolism, but low doses rarely have these effects. Combination of diuretics with beta-blockers is not recommended in patients with obesity or at high risk of diabetes because of adverse effects such as new-onset diabetes and adverse lipid metabolism. Thiazide-like diuretics such as chlorthalidone and indapamide have been found to be more effective than hydrochlorothiazide [133, 134, 138], but should be aware of hyponatremia or hypokalemia. Loop diuretics such as furosemide and torsemide are administered in the presence of CHF or when the GFR drops below 30 mL/min/1.73 m^2^ [135]. Spironolactone has proven effective in patients with heart failure and may be considered at low doses (25–50 mg) for treatment of resistant HTN.

##### Beta-blockers

Selective beta-1 blockers are recommended for patients with HTN in combination with angina pectoris, myocardial infarction, or tachycardia. Beta-blockers are also effective in younger patients who have higher heart rates [22]. However, they should be used with caution in patients with asthma, chronic obstructive pulmonary disease, second- or third-degree atrioventricular block, or peripheral vascular disease [22]. Beta-blockers can have adverse effects on blood glucose and lipid metabolism and should therefore be used cautiously in elderly patients or patients with elevated blood sugar, diabetes, or metabolic syndrome [139]. They should also be used carefully in patients with variant angina because they can worsen symptoms [140]. Because atenolol is inferior for stroke prevention, it is not recommended for elderly patients with HTN [141]. Concomitant use of beta-blockers and diuretics, which is not inferior in its BP-lowering effect, will increase the incidence of DM and should therefore be avoided in patients at high risk for developing DM [139]. Vasodilatory beta-blockers might have different effects than atenolol, but no comparative study has yet been performed to date [142, 143].

##### Calcium channel blockers

Long-acting calcium channel blockers are preferable to short-acting calcium channel blockers, which can cause tachycardia and increase cardiac workload. Because calcium channel blockers have a vasodilatory effect on the coronary artery, they are highly effective in patients with stable angina or variant angina, which is caused by coronary artery spasm. They are also effective for slowing the progression of carotid atherosclerosis and reducing cardiac hypertrophy [143]. The non-dihydropyridine calcium channel blockers, verapamil, and diltiazem, are effective after myocardial infarction because they do not produce reflex tachycardia. They are also effective in patients with hypertrophic cardiomyopathy because they improve diastolic filling. The common side effects of dihydropyridine calcium channel blockers are tachycardia, ankle edema, headache, and facial flushing. Non-dihydropyridine calcium channel blockers may cause constipation, conduction delay, and decreased myocardial contractility and should therefore be prescribed cautiously to patients with systolic heart failure or heart block. In addition, special caution is needed when administering them in combination with beta-blockers in elderly patients.

##### Angiotensin converting enzyme inhibitors / angiotensin receptor blockers

ACE inhibitors/ARBs reduce mortality in patients with heart failure and help to inhibit the progression of renal disease. They also help to prevent LVH and atherosclerosis and have little effect on blood glucose or lipid metabolism [144]. In addition, they can improve vascular endothelial cell function and promote vascular reverse remodeling. However, they can cause a hypotensive response in dehydrated or elderly patients [145]. When administered to a patient with bilateral renal artery stenosis, they can produce adverse effects such as severe hypotension and deterioration of renal function. The serum creatinine level may increase within the first two months after the start of treatment. However, there is no need to discontinue the drug unless the serum creatinine increases to less than 30% rise than the baseline creatinine level or unless serum potassium is 5.5 mEq/L or higher [146]. Care should be taken in patients with a serum creatinine level higher than 3.0 mg/dL [147]. The blood potassium level and renal function should be checked before and within 1–4 weeks after administration of the drug and then again three or six months later. ACE inhibitors inhibit bradykinin degradation and can thus cause a dry cough, but this resolves within a few days to a few weeks after stopping the medication. Dry cough is more common in women and non-smokers. ARBs have no effect on bradykinin and therefore rarely cause cough. ACE inhibitors/ARBs are contraindicated in pregnant women because of their teratogenic effects on the fetus.

Side effects include hyperkalemia, acute renal damage in bilateral renal artery stenosis, abnormal taste, leukopenia, angioedema, and rash.

##### Other agents

Alpha-blockers can alleviate urinary symptoms in patients with prostate enlargement and also improve the metabolism of glucose and lipids. However, they can cause orthostatic hypotension and are associated with worsening of heart failure. Agents that act on the central nervous system, such as clonidine, methyldopa, and reserpine, have many side effects and are therefore not recommended as first-line drugs. Renin inhibitors have been developed and used in other countries but were not introduced in Korea. Renin inhibitors significantly reduced BP and proteinuria when used alone or in combination with diuretics. However, aliskiren has not been proven to improve the prognosis of patients with CVD. Methyldopa is still preferred for the treatment of HTN in pregnant women but is not the first choice because of its side effects. Hydralazine is a vasodilator that is relatively safe for pregnant women with HTN.

#### Combination therapy

**Fig. 3:**
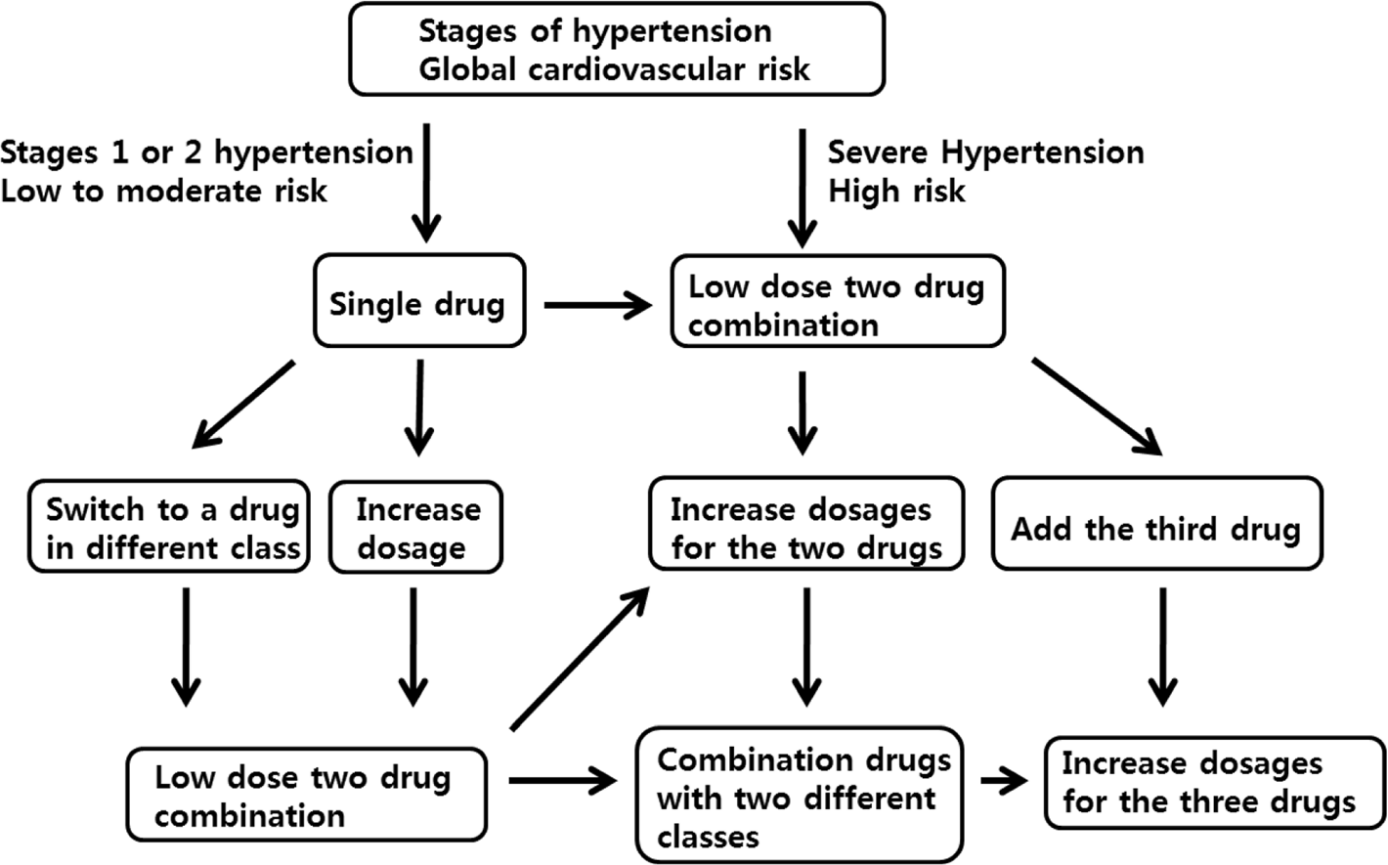
Choice of single drug or combination drugs according to the level of blood pressure and the global cardiovascular risk

More than 2/3 of patients with HTN require drugs from more than two drug classes with different mechanisms to achieve control of HTN. Combination therapy is particularly helpful for patients receiving prolonged BP treatment, high risk patients, and patients with lower target BP. If the first drug used is not effective for BP control, then a drug of another class should be tried. If the efficacy is insufficient, the dose should be increased or another drug added. However, it is recommended to combine two different drugs in smaller doses rather than to increase the dosage of one drug because such low dose combinations lower BP more effectively and improve the adherence while decreasing the adverse effects (Fig. 3) [148]. In high risk HTN, the prognosis can be improved as soon as BP is lowered below the target. Therefore, we recommend the use of combination therapy from the beginning in grade 2 or high risk HTN [129]. In this case, single pill combination can be considered as a first-line treatment because it improves drug adherence [149]. However, there is the disadvantage that the single pill combination cannot control the drug dose freely when the side effect of the drug occurs. Since there is no direct comparative study with the existing combination therapies, the choice of the single pill combination may be determined by adherence, possible adverse effects, and the target BP.

**Fig. 4:**
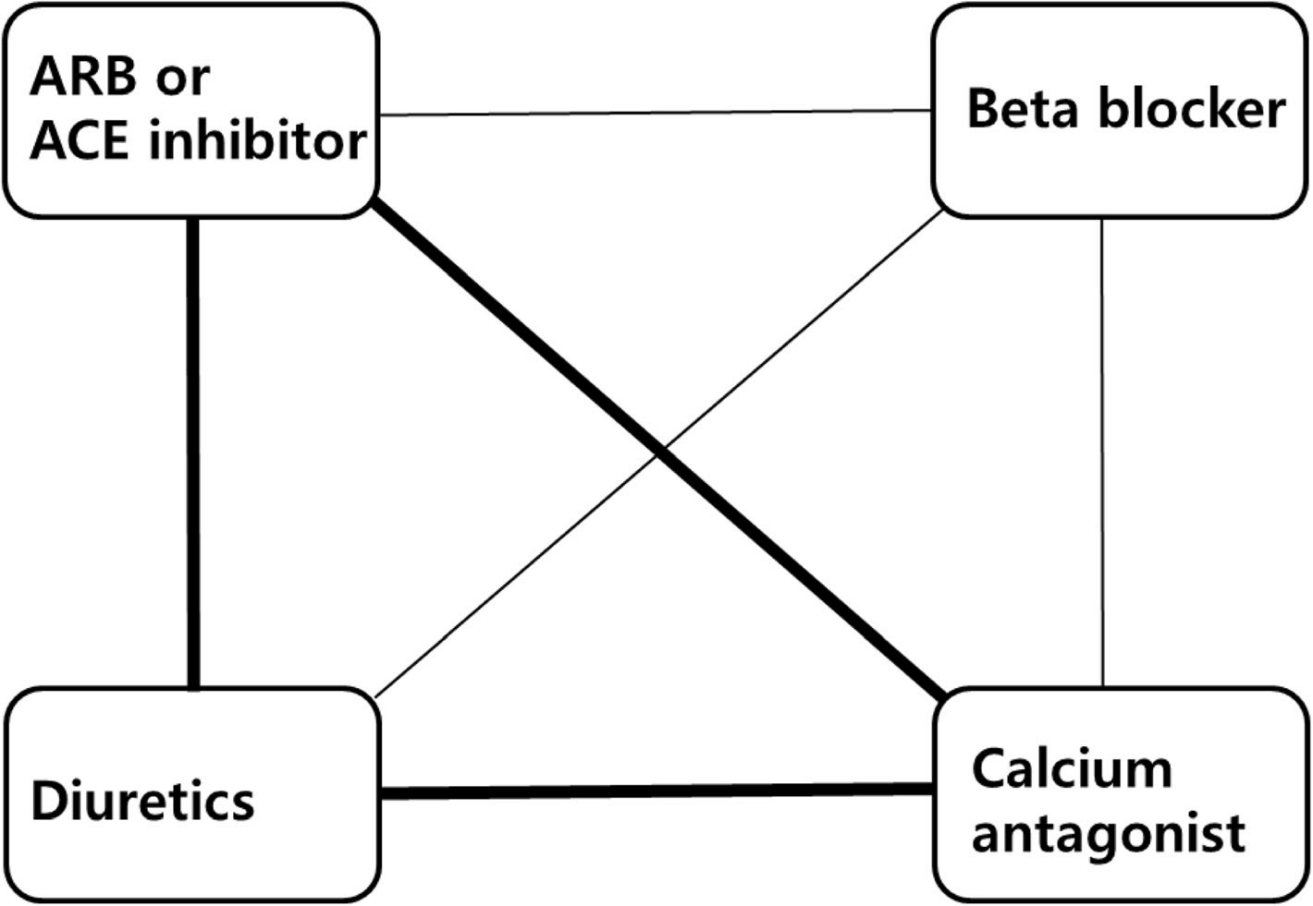
Recommended combination therapy, thick lines; preferred combination, thin line; feasible combination

If BP is not controlled with a single drug, two drugs should be combined for BP control. Combination therapy is more effective than single-drug therapy at a higher dose [148]. However, it has not been fully evaluated which combination is best. Combination therapy chosen from the renin-angiotensin system inhibitors, calcium channel blockers, and diuretics is recommended first because it has shown relatively good results [58, 65, 150], but beta-blockers can also be combined with drugs of other classes (Fig. 4). However, the combination of beta-blockers and diuretics may increase the incidence of diabetes and metabolic disorders and thus requires regular monitoring. Combination therapy with ARBs and ACE inhibitors may be slightly more effective for reducing proteinuria but increases the risk for end-stage renal failure, stroke, and other CVD, therefore is not recommended [151–153].

#### Resistant hypertension

**Table:** 

**Recommendations**	**Class**	**Level**	**References**
•Examination of adherence and ambulatory BP monitoring or home BP monitoring is recommended to exclude pseudo-resistant HTN.	**I**	**B**	[154]
• Addition of low-dose spironolactone can be considered for the treatment of resistant HTN.	**IIa**	**B**	[136]

Resistant HTN is defined as BP that cannot be controlled (BP ≥140/90 mmHg) despite treatment with more than three different classes of antihypertensive drugs, including diuretics. The prevalence of resistant HTN is reported to be 5–30% in other countries. However, considering the frequency of pseudo-resistant HTN, the prevalence of true resistant HTN is assumed to be below 10%. Patients with resistant HTN are at much higher risk for complications such as CVD and CKD [155].

Among the wide range of causes of resistant HTN (Table 13), non-adherence is the most common. In addition, medications taken for relief of cold symptoms, non-steroidal anti-inflammatory drugs, adrenal cortical steroids, birth control pills, excessive salt intake, and excessive alcohol consumption can also cause resistant HTN. If diuretics have not been included in the regimen, volume overload can cause resistant HTN. Finally, secondary HTN can cause resistant HTN.

**Table 13:** Differential diagnosis of uncontrolled hypertension

Causes	Conditions
Inappropriate BP measurement	White coat hypertension Calcified vessel in the elderly (pseudohypertension) Wrong cuff use, using too small cuff
Lifestyle factors	Severe weight gain, Heavy or binge drinking, Sleep apnea syndrome
Volume overload	Excess salt intake, Volume expansion by renal diseases, Insufficient use of diuretics
Medication	Poor compliance, Insufficient dose, or ineffective combination
Drug interaction/adverse effects	Nonsteroidal anti-inflammatory drugs (NSAIDs) Oral pills, Corticosteroid, Herbal licorice
Secondary hypertension	

To diagnose resistant HTN, treatment adherence should be confirmed and then the home BP or ambulatory BP should be monitored in order to exclude white coat HTN. If BP cannot be controlled despite the use of effective doses of three different classes of drug, then the dose of diuretics should be increased, or thiazide diuretics changed to loop diuretics in patients with renal impairment. However, most patients with resistant HTN require a different mechanism for BP control, and the fourth drug added should be spironolactone, amiloride, or an alpha-blocker such as doxazosin [156–159]. When spironolactone or amiloride is added in patients treated with ACE inhibitors or ARBs, the blood potassium level should be checked within 1–2 weeks.

#### Device-based hypertension treatment

Carotid baroreceptor stimulation or renal denervation may be tried in patients with true resistant HTN since the risk of procedure-related complication is low. However, there are non-responders and a lack of evidence of long-term effects, therefore it is not currently recommended [160, 161].

#### Reduction or discontinuation of antihypertensive medications

In patients whose BP has been well controlled for years, or for an extended period, it may be possible to reduce the number and/or dosage of drugs. This may particularly be the case if BP control is accompanied by healthy lifestyle changes such as weight loss, exercise habits, and a low-fat and low-salt diet, which remove environmental pressor influences. A reduction of medications should be made gradually, and the patient should be checked frequently, for example at least for three months, because reappearance of HTN can occur frequently [22].

#### Managing concomitant cardiovascular disease risk

The goal of antihypertensive therapy is to reduce the overall CV risk in patients who have other risk factors such as diabetes, dyslipidemia, CAD, stroke, and CKD. Accordingly, these other risk factors should be treated concurrently.

##### Antiplatelet therapy

Aspirin administration has been shown to produce an absolute benefit for the secondary prevention of CVD in patients with HTN [162]. However, the role of aspirin for primary prevention remains a matter of debate, leaning towards the negative side. Low-dose aspirin (100 mg) can be prescribed to patients in high risk groups in order to reduce the risk of CVD [162, 163]. Antiplatelet agents should be administered after the BP is controlled, and patients should be checked periodically for gastrointestinal bleeding.

##### Lipid-lowering agents

Lipid-lowering agents have a protective effect on high risk patients with HTN. Although there is very little Korean data, a 50% reduction in LDL-cholesterol in patients who had an LDL-cholesterol level ≥ 130 mg/dL significantly lowered the risk of CVD [164]. Lowering the LDL-cholesterol level to < 70 mg/dL should be recommended in patients with CAD [165]. There is evidence for reducing the LDL-cholesterol level to < 135 mg/dL in patients with stroke [166], however, there is little data regarding the effects of lowering the LDL-cholesterol to < 70 mg/dL in such patients.

##### Glycemic control

The goal of glycemic control in patients with DM is less than 6.5% of glycated hemoglobin, further lowering if there is no complication associated with early diabetes and low risk of hypoglycemia. Conversely, in patients with severe hypoglycemia, short life expectancy, advanced microvascular and macrovascular complications, and elderly people over 75 years of age, the goal of glycemic control can be individualized taking into account the risk of hypoglycemia [167].

#### Patient monitoring and follow-up

Patients should generally be followed up once monthly, at least until the target BP is achieved. Patients with severe HTN (grade 2) require more frequent follow-up. The serum potassium and creatinine levels should be measured at least 1–2 times yearly. If the BP is controlled and stable, then the patient should be followed up every 3–6 months. A longer follow-up interval may be associated with low adherence. Therefore, patient adherence also must be monitored as well as the regular follow up tests for blood samples. A longer follow-up interval to monitor the status of BP control can be achieved by encouraging home BP measurement.

#### Adherence

Trust between doctor and patient is the most important issue in the treatment of HTN, and the patient should therefore be encouraged to participate in the treatment plan. Many patients may have obtained information about various antihypertensive agents through various routes, so discussion may be necessary. First, identify the patient’s point of view to determine the relative importance of efficacy, cost-effectiveness, and side effects. It is necessary to reduce overall CV risk as much as possible while maintaining the patient’s adherence. Self-measurement of BP by using HBPM can improve adherence [5].

## Data Availability

Not applicable.
